# A Two-Stage Culture Method for Zygotic Embryos Effectively Overcomes Constraints Imposed by Hypocotyl and Epicotyl Seed Dormancy in *Paeonia ostii* ‘Fengdan’

**DOI:** 10.3390/plants8100356

**Published:** 2019-09-20

**Authors:** Xiuxia Ren, Ya Liu, Byoung Ryong Jeong

**Affiliations:** 1 Division of Applied Life Science (BK21 Plus Program), Graduate School, Gyeongsang National University, Jinju 52828, Korea; xiuxia0823@163.com (X.R.); liuya113@mails.ucas.ac.cn (Y.L.); 2 Institute of Agriculture and Life Science, Gyeongsang National University, Jinju 52828, Korea; 3 Research Institute of Life Sciences, Gyeongsang National University, Jinju 52828, Korea

**Keywords:** *Paeonia ostii* ‘Fengdan’, embryo culture, dormancy-breaking, hypocotyl, epicotyl, PGR, temperature, light quality

## Abstract

The effect of the exogenous hormone and light quality on breaking hypocotyl and epicotyl dormancy was studied. The results showed that the greatest percentage of hypocotyl dormancy breaking was observed with the Murashige and Skoog (MS) medium supplemented with or without 1.0 mg·L^−1^ gibberellin 3 (GA_3_), while ABA and endosperm greatly inhibited hypocotyl dormancy breaking. This suggests that hypocotyl dormancy of the *Paeonia ostii* ‘Fengdan’ embryo could be easily overcome by removing constraints of the surrounding endosperm, and ABA may be one of the constraint factors contained in the endosperm. The percentage of epicotyl dormancy breaking was also greatly affected by the concentration of 6-benzylaminopurine (BA) and GA_3_. Compared to BA by itself, adding GA_3_ to the medium containing BA highly enhanced epicotyl dormancy breaking, with the greatest percentage of epicotyl dormancy breaking in MS medium supplemented with both 0.5 mg·L^−1^ BA and 0.5–1.0 mg·L^−1^ GA_3_. The percentage of hypocotyl and epicotyl dormancy breaking was also affected by light and its quality. Red light-emitting diodes (LEDs) had the same effect as a dark condition on the hypocotyl dormancy breaking, while blue LEDs and a combination of red and blue LEDs had a negative effect on the hypocotyl dormancy breaking. Unexpectedly, blue LEDs greatly enhanced, whereas red LEDs inhibited, epicotyl dormancy breaking. Conclusively, a two-stage culture method was recommended for breaking the hypocotyl and epicotyl dormancy: hypocotyl dormancy was broken first using the MS medium without any plant growth regulators in the dark (25 °C), and epicotyl dormancy was subsequently broken with the MS medium supplemented with both 1.0 mg·L^−1^ GA_3_ and 0.5 mg·L^−1^ BA under blue light.

## 1. Introduction

*Paeonia ostii* ‘Fengdan’, a species of tree peony (*Paeonia*, Sect. Moutan DC.), is a famous medicinal plant, the roots of which are widely found in Chinese traditional medicine [1,2]. Besides, *Paeonia ostii* ‘Fengdan’ is also an important parent plant for breeding new varieties [3]. Seeds of *Paeonia ostii* ‘Fengdan’ are rich in unsaturated acid, especially α-linolenic acid (ALA), and the oil produced from tree peony seed is now very popular in China [4,5]. *Paeonia ostii* ‘Fengdan’ is one of the most widely used tree peony species for oil extraction due to its great setting percentage and high level of unsaturated content [6].

Propagation by seeds is an important and widely used method for tree peonies, and it is also required for the breeding of new cultivars [5]. However, the seeds of the tree peony have deep epicotyl dormancy as well as hypocotyl dormancy [7,8], which is an adaptation that resulted from biological evolution to help seeds survive in adverse environments [9]. It normally takes more than 8–9 months for tree peony seeds to germinate without any treatment under natural conditions, and the survival percentage of the seedlings is very low, which delays the propagation process and has restricted the development of tree peony production [7,10]. Therefore, seed dormancy breaking has been a hot topic for scientific research on the tree peony for decades [8,11,12]. The methods for breaking seed dormancy of the tree peony include exogenous gibberellin treatment, chilling sand treatment, and their combinations with specific gibberellin concentrations, chilling sand treatment temperatures, and treatment periods, respectively [7,13]. It has been fully proven that hypocotyl and epicotyl dormancy of the tree peony should be broken separately using with different treatments [7,8].

Seed dormancy is considered as an obstacle to seed germination [14], an adaptation that resulted from biological evolution to help seeds survive in adverse environments [9], and as a seed characteristic that could determine the conditions required for germination [15,16]. Therefore, by definition, any environmental condition or treatment that changes the conditions required for germination alter the dormancy [17,18]. It is widely accepted that temperature and plant hormones could regulate both the dormancy and germination [16,19,20,21]. Gibberellin and abscisic acid play an important role in seed germination [22]. Light has both been considered to stimulate germination [16] and to terminate dormancy [23]. Therefore, chilling sand treatment, exogenous gibberellin, and lights of special quality are widely used in seed dormancy breaking. The dormancy-breaking process, especially chilling sand treatment, incurs large economic and labor costs [11].

Embryo culture is a promising method to accelerate seed dormancy breaking. Moreover, it can be used to overcome embryo abortion and dysplasia, shorten the period of seed dormancy, and enhance germination [24]. Some research on embryo culture of *Paeonia* plants has been published [25,26,27]. Shoots or callus were induced first [25,27], and then roots were produce to make whole plants [26,28]. The whole process always lasted more than 3–4 months [28] and there are still some problems, such as rooting quality and browning. In this study, embryo culture and the seed dormancy-breaking process followed the natural rules to solve the above problems. The hypocotyl was broken first and thereafter the epicotyl was broken. The effect of the culture medium, exogenous hormone, and the light and its quality on breaking hypocotyl and epicotyl dormancy was analyzed to define an optimal medium and condition for dormancy breaking and generating new plantlets in a short time using the tissue culture method.

## 2. Materials and Methods

### 2.1. Plant Materials and Sterilization

Seeds of *P. ostii* ‘Fengdan’ were used in this study. Disease-free seeds were thoroughly washed in running tap water for 12 h and soaked in 200 mg·L^−1^ gibberellin 3 (GA_3_) for 18 h. The surface of the pretreated seeds was sterilized in 3% sodium hypochlorite (NaClO) for 5 minutes, and in 70% ethanol for 5 minutes, followed by eight rinses with sterilized deionized water. Seed coats of the sterilized seeds were removed carefully. The endosperm part of seeds was discarded and the embryos were used as the explants. Besides, those zygotic embryos with the endosperm were also used as explants for further research. The longitudinal and transverse lengths of the embryos and whole seeds were measured.

### 2.2. Hypocotyl and Epicotyl Dormancy Breaking as Affected by GA_3_, ABA, and Endosperm

**Figure 1:**
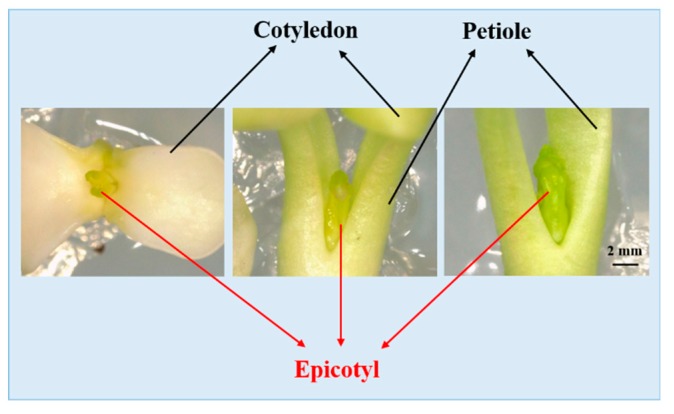
The structure of embryos viewed as losing the epicotyl dormancy. The scale bar is 2 mm.

The solid Murashige and Skoog (MS, 1962) medium containing 3% (w/v) sucrose and 0.80% (w/v) agar were used in all treatments. Zygotic embryos were cultured on the MS medium without any plant growth regulators (PGRs), MS medium supplemented with 1.0 mg·L^−1^ gibberellin 3 (GA_3_), MS medium supplemented with 1.0 mg·L^−1^ ABA, and MS medium supplemented with both 1.0 mg·L^−1^ GA_3_ and 1.0 mg·L^−1^ ABA at 25 °C. Zygotic embryos with endosperm were cultured on the MS medium supplemented with 1.0 mg·L^−1^ GA_3_. The pH of all media used was adjusted to 5.80 before autoclaving at 121 °C for 15 minutes. All zygotic embryos, with or without endosperm, were cultured in dark for 2 weeks and subsequently cultured under a white light with a photoperiod of 16 h, a light intensity of 50 μmol·m^−2^·s^−1^ photosynthetic photon flux density (PPFD), and a day/night temperature of 24/18 °C. The percentage of hypocotyl and epicotyl dormancy breaking refers to the number of embryos that release hypocotyl or epicotyl dormancy dividing by the whole number of embryos according to the following formulas. The percentage of hypocotyl and epicotyl dormancy breaking was recorded after 4 weeks of culture. Embryos are viewed as losing their hypocotyl dormancy when hypocotyls are more than three times the initial length of the embryo. Epicotyl dormancy is thought as being removed when the epicotyls stretched out between two petioles of two cotyledon (at the base of the petioles), which can be seen by eye (Figure 1).

(1) The percentage of hypocotyl dormancy breaking = number of embryos that release hypocotyl dormancy/the whole number of embryos
(2) The percentage of epicotyl dormancy breaking = number of embryos that release epicotyl dormancy/the whole number of embryos

### 2.3. Hypocotyl and Epicotyl Dormancy Breaking as Affected by GA_3_ and 6-Benzylaminopurine (BA)

Zygotic embryos were cultured on the MS medium supplemented with GA_3_ (0, 0.5, 1.0, 2.0, or 3.0 mg·L^−1^) by itself or in combination with 6-benzylaminopurine (BA; 0, 0.5, and 1.0 mg·L^−1^) at 25 °C. The culture condition and culture period were same with that of Section 2.2. The percentage of hypocotyl and epicotyl dormancy breaking was recorded after 4 weeks of culture.

### 2.4. Hypocotyl and Epicotyl Dormancy Breaking as Affected by BA and GA_3_ in a Two-Stage Culture Method

There were eight treatments in this experiment, numbered 1–8 (Table 1). In treatments 1–5, embryos were cultured on the MS medium without PGRs at 25 °C in the first culture stage, and were subsequently cultured on the MS medium without PGRs at 25 °C (No. 1), MS medium supplemented with 1.0 mg·L^−1^ GA_3_ at 25 °C (No. 2), MS medium supplemented with 0.5 mg·L^−1^ BA at 25 °C (No. 3), and MS medium supplemented with 0.5 mg·L^−1^ BA and 1.0 mg·L^−1^ GA_3_ at 25 °C (No. 4) in the second culture stage. Embryos in treatments 5 were cultured on the MS medium supplemented with 1.0 mg·L^−1^ GA_3_ at 25 °C in the two culture stages (No. 5). In treatment 6, embryos were cultured on the MS medium supplemented with 0.5 mg·L^−1^ BA and 1.0 mg·L^−1^ GA_3_ at 25 °C in the two culture stages. All zygotic embryos were cultured in dark during the first culture stage (4 weeks) and were subsequently cultured under a white light with a photoperiod of 16 h, a light intensity of 50 μmol·m^−2^·s^−1^ PPFD, and a day/night temperature of 24/18 °C in the second culture stage (4 weeks). The percentages of hypocotyl and epicotyl dormancy breaking were measured after 8 weeks. Regular subcultures were carried out every four weeks.

### 2.5. Hypocotyl and Epicotyl Dormancy Breaking as Affected by the Light and Its Quality

MS medium containing 3% (w/v) sucrose and 0.80% (w/v) agar without PGRs were used in all treatments. Embryos were cultured in dark, or under red light-emitting diodes (LEDs, custom-made, SungKwang LED, Incheon, Republic of Korea), blue LEDs (custom-made, SungKwang LED, Incheon, Republic of Korea), or a combination of red and blue LEDs (custom-made, SungKwang LED, Incheon, Republic of Korea) for 8 weeks with a photoperiod of 16 h, a light intensity of 50 μmol·m^−2^·s^−1^ PPFD, and a day/night temperature of 24/18 °C (except for the dark condition). The percentage of dormancy breaking of the hypocotyl and epicotyl was recorded after 8 weeks. Regular subcultures were carried out every four weeks.

### 2.6. Statistical Analysis

In this study, each treatment has three replicates with 20 explants per replicate. Each replicate includes four Petri dishes and there were five explants per petri dish. The data are presented as the mean ± standard error. All data were analyzed using one-way ANOVA, and the means were separated using the Duncan’s multiple range test at *p* ≤ 0.05.

## 3. Results

### 3.1. The Effect of GA_3_, ABA, and Endosperm on Hypocotyl Dormancy Breaking

Seeds of *P. ostii* ‘Fengdan’ were round and full and seed surface had a bright and shiny black color. The longitudinal and transverse length of embryo is about 1.5 mm and 1.0 mm, respectively, while the longitudinal and transverse length of whole seeds is about 10 mm and 9.5 mm. Hypocotyl dormancy breaking was largely affected by the GA_3_, ABA, and endosperm (Table 2). Hypocotyl dormancy breaking was strongly inhibited by the ABA and endosperm. The percentage of hypocotyl dormancy breaking was the greatest with the MS medium supplemented either with or without 1.0 mg·L^−1^ GA_3_ at 25 °C. Thereby, MS medium without PGRs at 25 °C is recommended for breaking the hypocotyl dormancy, which had similar effect on breaking the hypocotyl dormancy as the MS medium supplemented with 1.0 mg·L^−1^ GA_3_ at 25 °C.

### 3.2. The Effect of GA_3_ and BA Concentration on Hypocotyl and Epicotyl Dormancy Breaking

**Figure 2:**
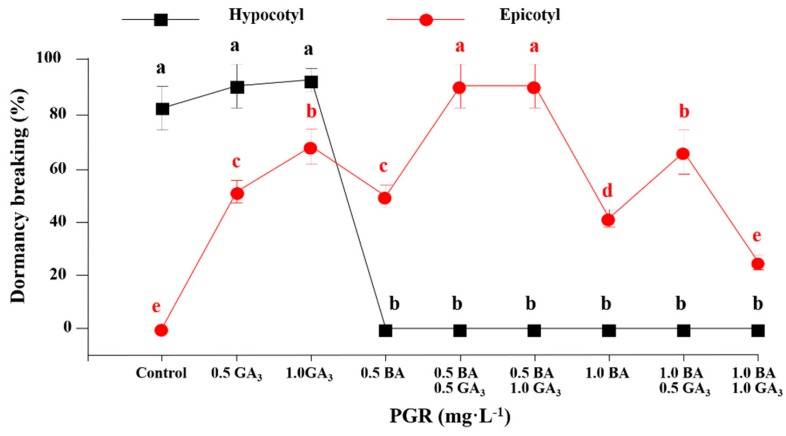
The percentage of epicotyl dormancy breaking affected by the BA and GA_3_ concentration after 8 weeks of culture. The PGR concentration is expressed in mg·L^−1^. The MS medium without any PGRs was used as the control. Different letters indicate separation among treatments by Duncan’s multiple range test at a 5% level.

**Figure 3:**
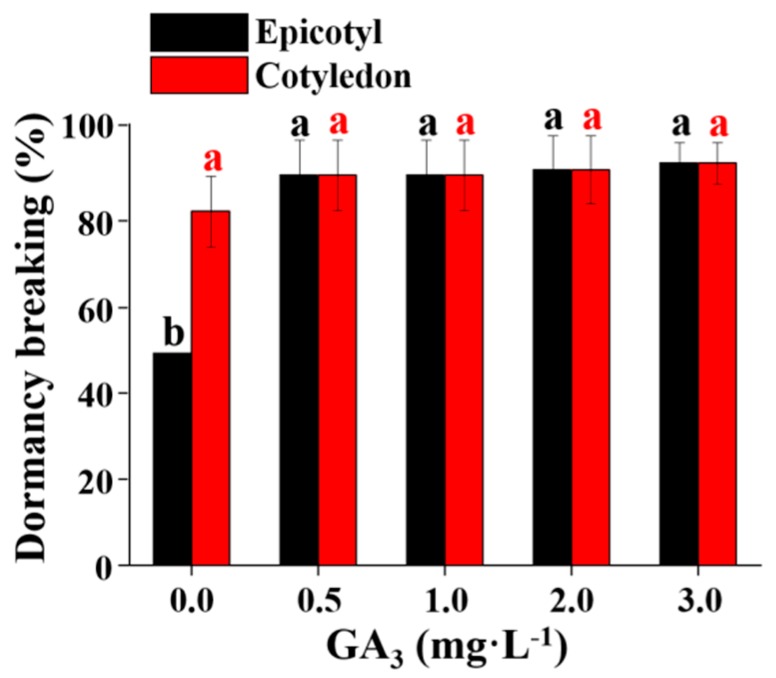
The percentage of epicotyl dormancy breaking affected by the GA_3_ concentration. Different letters indicate separation among treatments by Duncan’s multiple range test at a 5% level.

Hypocotyl and epicotyl dormancy breaking were strongly influenced by the concentration of BA or GA_3_. The percentage of hypocotyl dormancy breaking was high in the MS medium without PGRs and in the MS medium supplemented with 0.5 or 1.0 mg·L^−1^ GA_3_, whereas it was zero when BA was added to the MS medium. Both BA and GA_3_ can accelerate the breaking of epicotyl dormancy. The percentage of epicotyl dormancy breaking was significantly enhanced with the combination of 0.5 mg·L^−1^ BA and 0.5–1.0 mg·L^−1^ GA_3_ (Figure 2). The percentage of epicotyl dormancy breaking was highly increased when GA_3_ was added to the MS medium. However, different concentrations (0.5, 1.0, 2.0, or 3.0 mg·L^−1^) of GA_3_ had the same effect on the epicotyl dormancy breaking (Figure 3).

### 3.3. Hypocotyl and Epicotyl Dormancy Breaking Affected by GA_3_ and BA in a Two-stage Culture Method

The percentage of hypocotyl dormancy breaking was high in the MS medium supplemented with or without 1.0 mg·L^−1^ GA_3_ at 25 °C in the first culture stage, which dramatically decreased to zero when BA was added (Table 3). For the second culture stage, the percentage of epicotyl dormancy breaking was the lowest in the MS medium without PGRs at 25 °C (control) (Table 3). Furthermore, the percentage of epicotyl dormancy breaking in the MS medium supplemented with BA or GA_3_ (25 °C) was greater than it was in the MS medium without PGRs (25 °C). A combination of BA and GA_3_ was much more effective than BA or GA_3_ alone in breaking epicotyl dormancy. Therefore, the greatest percentage of epicotyl dormancy breaking was found in the MS medium supplemented with 1.0 mg·L^−1^ GA_3_ and 0.5 mg·L^−1^ BA. In conclusion, a two-stage culture method was developed for breaking hypocotyl and epicotyl dormancy: hypocotyl dormancy should be broken first by using the MS medium (25 °C) and epicotyl dormancy could subsequently be broken with the MS medium supplemented with 1.0 mg·L^−1^ GA_3_ and 0.5 mg·L^−1^ BA (25 °C).

### 3.4. The Effect of the Light and Its Quality on Breaking Hypocotyl and Epicotyl Dormancy

**Figure 4:**
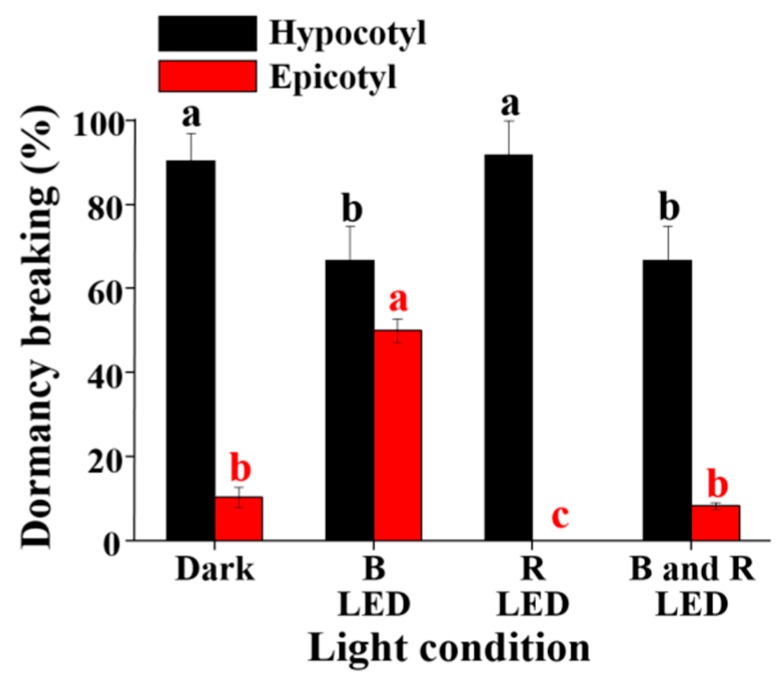
The percentage of hypocotyl and epicotyl dormancy breaking as affected by the concentration of BA and GA_3_. Different letters indicate separation among treatments by Duncan’s multiple range test at a 5% level.

The percentage of hypocotyl and epicotyl dormancy breaking was largely affected by the light and its quality. The percentage of hypocotyl dormancy breaking was significantly lower with blue LEDs and a mix of blue and red LEDs than it was in the dark (the control) or with red LEDs, indicating that blue LEDs inhibit hypocotyl dormancy breaking (Figure 4). Interestingly, red and blue LEDs have the opposite effect on epicotyl dormancy breaking. The percentage of epicotyl dormancy breaking was zero with red light, which was significantly lower than that with darkness (the control). The percentage of epicotyl dormancy breaking was significantly enhanced with blue LEDs compared to that with darkness (the control), red LEDs, and a mix of blue and red LEDs. In short, blue LEDs inhibited hypocotyl dormancy breaking but accelerated epicotyl dormancy breaking. Red LEDs slightly accelerated hypocotyl dormancy breaking, and the effect was similar with darkness. Inversely, red LEDs inhibited epicotyl dormancy breaking, suggesting that darkness or red LEDs could be used for hypocotyl dormancy breaking and blue LEDs could be utilized for epicotyl dormancy breaking.

## 4. Discussion

Seed dormancy and germination could be largely affected by the temperature, light, and application of exogenous hormones [29,30,31,32,33]. In this study, the effects of the light and exogenous hormone on breaking the hypocotyl and epicotyl dormancy were studied using in vitro cultured embryos as the material. The results showed that embryos grew in size and hypocotyl dormancy was broken in the MS medium supplemented with or without GA_3_ at 25 °C after a period of time for embryo development, suggesting that hypocotyl dormancy of the *Paeonia ostii* ‘Fengdan’ embryo could be easily overcome in very short time by removing constraints of the surrounding endosperm. Hypocotyl dormancy of *Paeonia ostii* ‘Fengdan’ and *Asarum canadense* seeds was broken gradually after a long period of culture at warm temperature without any other treatments [8,34]. Seed dormancy is categorized into physiological dormancy (PD), morphological dormancy (MD), morphophysiological dormancy (MPD), physical dormancy (PY), and combinational dormancy (PY + PD). Embryos of MD seeds are very small and underdeveloped [15,19,35], and they just need time to grow and germinate. Embryos of MPD seeds are underdeveloped, and there are also some physiological components of MPD seeds in their dormancy [15,19]. Seeds with MPD can only lose physiological seed dormancy with dormancy-breaking treatments such as cold stratification, warm stratification, or wet/dry cycling after ripening, and then grow and develop within the seed prior to radicle emergence [10,15,19]. The endosperm as well as exogenous ABA inhibited dormancy breaking of *P. ostii* ‘Fengdan’, indicating that the endosperm may contain physiological inhibitors and ABA could be one of physiological inhibitors for dormancy breaking. The tree peony has both hypocotyl and epicotyl dormancy [10]. After ripening, cold stratification is required in breaking hypocotyl and epicotyl dormancy of tree peony seeds, which has been proved in most *Paeonia* species [7]. Those embryos with extended hypocotyls could not directly produce the epicotyls or shoots without any treatment [36]. In other words, dormancy breaking of epicotyls requires physiological dormancy-breaking treatment, such as cold exposure or exogenous hormone application instead [36]. Besides, the embryo of *Paeonia* species is very small and underdeveloped, and it needs to grow within seed before the radicle emerges [7]. We also found that the embryo of *P. ostii* ‘Fengdan’ is very small, as the longitudinal and transverse lengths of embryo are only about 1.5 mm and 1.0 mm, respectively, about 7 and 10 times of that of the whole seeds. Therefore, the dormancy of tree peony seeds is thought to be a MPD seed according to the rule of Baskin and Baskin [19]. 

Interestingly, an exogenous BA had a different role in breaking hypocotyl and epicotyl dormancy. It inhibited hypocotyl elongation and accelerated epicotyl dormancy breaking. It is reported that cytokinins could inhibit hypocotyl elongation in *Arabidopsis* [37,38]. Some research also shows that cytokinins, together with gibberellins or brassinosteroids, positively regulated the seed dormancy breaking process in *Orobanche*, *Lepidium sativum* (cress), and lettuce [21,39]. In contrast to exogenous cytokinin, exogenous gibberellin promotes seed dormancy breaking in many species, such as *Arabidopsis*, tomato, lettuce, and tobacco [40,41,42,43,44], which was also seen with epicotyl dormancy breaking of *P. ostii* ‘Fengdan’ in our study. Gibberellin and cytokinin generally promote dormancy breaking [45]. We found that a combination of BA and GA_3_ was the most effective in breaking epicotyl dormancy, which was much better than with either BA or GA_3_ alone. Exogenous BA and GA_3_ positively affected epicotyl germination in herbaceous peony as well [25]. Low temperature (4 °C) could accelerate seed dormancy breaking and improve the germination percentage [46]. Gibberellin biosynthesis genes are positively regulated by low temperature [46]. This also explains why a low temperature treatment could be replaced by gibberellin for dormancy breaking [15]. Epicotyl dormancy breaking of *P. ostii* ‘Fengdan’ was highly accelerated by the application of a combination of exogenous BA and GA_3_.

Light is also vital for seed germination in *Arabidopsis* [31]. The most active dormancy-breaking light is red light, and far-red light inhibits dormancy breaking [47]. Phytochrome is the red and far-red light photoreceptor, which could also regulate the biosynthesis of gibberellic acid [44]. Red light induces, but far-red light inhibits, seed dormancy breaking [48]. Dark stratification is often used to alleviate physiological dormancy in *Lolium rigidum* and *Eragrostis curvula* [49,50]. We also found that hypocotyl dormancy breaking was enhanced by dark condition and red light; however, the epicotyl dormancy was slightly suppressed by red light. Furthermore, blue light also greatly affected the dormancy breaking of *P. ostii* ‘Fengdan’. Hypocotyl dormancy breaking of *P. ostii* ‘Fengdan’ was inhibited by blue light, where the percentage of hypocotyl dormancy breaking was significantly lower under blue LEDs than in the control (darkness), while epicotyl dormancy breaking of *P. ostii* ‘Fengdan’ was accelerated and enhanced by blue light. It is also found that blue light inhibits hypocotyl elongation in *Arabidopsis* [51] and wheat grain [52]. White and blue light trigger dormancy in cereal grains by promoting the expression of an ABA biosynthetic gene [53]. Dormant seeds of *L. rigidum* remain dormant in the light, but light stimulates seed germination after seeds lose dormancy through dark-stratification [54]. It is reported that light promotes germination or triggers dormancy, depending on the species [48].

Based on these results, we clearly found that the condition and treatment required for breaking hypocotyl and epicotyl dormancy are quite different, which means that hypocotyl and epicotyl dormancy should be broken in two stages using the optimal conditions and treatment for each stage. Therefore, we built up an effective culture method with two stages for the dormancy breaking of *P. ostii* ‘Fengdan’: hypocotyl dormancy was broken in the first stage with the MS medium supplemented with or without GA_3_ in dark at 25 °C; the epicotyl dormancy was subsequently broken with the MS medium supplemented with BA and GA_3_ under blue light at 25 °C.

## 5. Conclusions

In conclusion, an effective two-stage culture method was built up to break the hypocotyl and epicotyl dormancy. Hypocotyl dormancy was broken first using the MS medium in the dark, and epicotyl dormancy was subsequently broken with the MS medium supplemented with 1.0 mg·L^−1^ GA_3_ and 0.5 mg·L^−1^ BA under blue light.

## Figures and Tables

**Table 1:** The plant growth regulator (PGR) and temperature in the two-stage culture method for breaking the hypocotyl and epicotyl dormancy. Zygotic embryos were cultured for 4 weeks each in the first and second culture stages.

First Culture Stage			Second Culture Stage			Abbreviation
PGR (mg·L^−1^)		Temperature (°C)	PGR (mg·L^−1^)		Temperature (°C)	
GA_3_	BA		GA_3_	BA		
0.0	0.0	25	0.0	0.0	25	Control (no PGR, 25 °C)
0.0	0.0	25	1.0	0.0	25	Control → 1.0 GA_3_ (25 °C)
0.0	0.0	25	0.0	0.5	25	Control → 0.5 BA (25 °C)
0.0	0.0	25	1.0	0.5	25	Control → 1.0 GA_3_ and 0.5 BA (25 °C)
1.0	0.0	25	1.0	0.0	25	1.0 GA_3_ (25 °C)
1.0	0.5	25	1.0	0.5	25	1.0 GA_3_ and 0.5 BA (25 °C)

**Table 2:** The effect of GA_3_, ABA, and endosperm on hypocotyl dormancy breaking. MS: Murashige and Skoog.

Explant	Temperature (°C)	Medium	PGR (mg·L^−1^)		Hypocotyl Dormancy Breaking (%)
			GA_3_	ABA	
Embryo	25	MS	0.0	0.0	80.0 ± 2.1 a ^z^
Embryo	25	MS	1.0	0.0	81.7 ± 3.2 a
Embryo	25	MS	0.0	1.0	0.0 ± 0.0 b
Embryo	25	MS	1.0	1.0	0.0 ± 0.0 b
Embryo and endosperm	25	MS	1.0	0.0	0.0 ± 0.0 b
-	-	-	-	-	***

^z^ Mean separation within each column by Duncan’s multiple range test at a 5% level. ***Mean significant at *p* ≤ 0.001.

**Table 3:** The effect of the BA and GA_3_ on epicotyl dormancy breaking in a two-stage culture method.

Treatments (mg·L^−1^)	Dormancy Breaking (%)	
	Hypocotyl	Epicotyl
Control (no PGR, 25 °C)	80.0 ± 2.1 a ^z^	10.3 ± 0.8 d
Control → 1.0 GA_3_ (25 °C)	80.0 ± 2.1 a	30.4 ± 2.2 b
Control → 0.5 BA (25 °C)	80.0 ± 2.1 a	30.1 ± 1.8 b
Control → 1.0 GA_3_ and 0.5 BA (25 °C)	80.0 ± 2.1 a	92.5 ± 3.8 a
1.0 GA_3_ (25 °C)	80.0 ± 3.2 a	30.0 ± 2.0 b
1.0 GA_3_ and 0.5 BA (25 °C)	0.0 ± 0.0 b	91.7 ± 1.9 a
F-test	***	***

^z^ Mean separation within each column by Duncan’s multiple range test at 5% level. *** Mean significant at *p* ≤ 0.001.

## References

[1] Zhang H.F., Li X.F., Wu K., Wang M.K., Liu P., Wang X.S., Deng R.X. (2017). Antioxidant activities and chemical constituents of flavonoids from the flower of *Paeonia ostii*. Molecules.

[2] Wang Y.J., Dong C.L., Xue Z.Y., Jin Q.J., Xu Y.C. (2016). De novo transcriptome sequencing and discovery of genes related to copper tolerance in *Paeonia ostii*. Gene.

[3] Han X., Cheng F., Xiao J., Wang Y., Zhang D., Wang Y., Zhong Y. (2014). Crosses of Paeonia ostii ‘Feng Dan Bai’ as maternal parents and an analysis on the potential in tree peony breeding. J. Beijing For. Univ..

[4] Xie L.H., Niu L.X., Zhang Y.L., Jin M., Ji D., Zhang X.X. (2017). Pollen sources influence the traits of seed and seed oil in *Paeonia ostii* ‘Feng Dan’. HortScience.

[5] Cui H.L., Cheng F.Y., Peng L.P. (2016). Determination of the fatty acid composition in tree peony seeds using near-infrared spectroscopy. J. Am. Oil Chem. Soc..

[6] Peng L.P., Cai C.F., Zhong Y., Xu X.X., Xian H.L., Cheng F.Y., Mao J.F. (2017). Genetic analyses reveal independent domestication origins of the emerging oil crop *Paeonia ostii*, a tree peony with a long-term cultivation history. Sci. Rep..

[7] Zhang K., Yao L., Zhang Y., Baskin J.M., Baskin C.C., Xiong Z., Tao J. (2019). A review of the seed biology of *Paeonia* species (Paeoniaceae), with particular reference to dormancy and germination. Planta.

[8] Ren X.X., Xue J.Q., Wang S.L., Xue Y.Q., Zhang P., Jiang H.D., Zhang X.X. (2018). Proteomic analysis of tree peony (*Paeonia ostii* ‘Feng Dan’) seed germination affected by low temperature. J. Plant Physiol..

[9] Porceddu M., Mattana E., Pritchard H.W., Bacchetta G. (2015). Sequential temperature control of multi-phasic dormancy release and germination of *Paeonia corsica* seeds. J. Plant Ecol..

[10] Yu X., Zhao R., Cheng F. (2014). Seed germination of tree and herbaceous peonies: A mini-review. Seed Sci. Biotechnol..

[11] Xue J.Q., Wang S.L., Zhang P., Zhu F.Y., Ren X.X., Liu C.J., Zhang X.X. (2015). On the role of physiological substances, abscisic acid and its biosynthetic genes in seed maturation and dormancy of tree peony (*Paeonia ostii* ‘Feng Dan’). Sci. Hortic..

[12] Jing X., Zheng G. (1999). The characteristics in seed germination and dormancy of four wild species of tree peonies and their bearing on endangerment. Acta Phytophysiol. Sin..

[13] Cheng F.Y., Du X.J. (2008). Effects of chilling and gibberellic acid on the seed germination and seedling growth in *Paeonia ostii* ‘Feng Dan’. Acta Hortic. Sin..

[14] Bewley J.D. (1997). Seed germination and dormancy. Plant Cell.

[15] Finch-Savage W.E., Leubner-Metzger G. (2006). Seed dormancy and the control of germination. New Phytol..

[16] Vleeshouwers L., Bouwmeester H., Karssen C. (1995). Redefining seed dormancy: An attempt to integrate physiology and ecology. J. Ecol..

[17] Finch-Savage W.E., Cadman C.S.C., Toorop P.E., Lynn J.R., Hilhorst H.W.M. (2007). Seed dormancy release in *Arabidopsis* Cvi by dry after-ripening, low temperature, nitrate and light shows common quantitative patterns of gene expression directed by environmentally specific sensing. Plant J..

[18] Bentsink L., Koornneef M. (2008). Seed dormancy and germination. Arabidopsis Book.

[19] Baskin J.M., Baskin C.C. (2004). A classification system for seed dormancy. Seed Sci. Res..

[20] Koornneef M., Bentsink L., Hilhorst H. (2002). Seed dormancy and germination. Curr. Opin. Plant Biol..

[21] Kucera B., Cohn M.A., Leubner-Metzger G. (2005). Plant hormone interactions during seed dormancy release and germination. Seed Sci. Res..

[22] Seo M., Nambara E., Choi G., Yamaguchi S. (2008). Interaction of light and hormone signals in germinating seeds. Plant Mol. Biol..

[23] Benech-Arnold R.L., Sánchez R.A., Forcella F., Kruk B.C., Ghersa C.M. (2000). Environmental control of dormancy in weed seed banks in soil. Field Crops Res..

[24] Raghavan V. (2003). One hundred years of zygotic embryo culture investigations. In Vitro Cell Dev. Biol. Plant.

[25] Buchheim J.A.T., Burkhart L.F., Meyer M.M. (1994). Effect of exogenous gibberellic acid, abscisic acid, and benzylaminopurine on epicotyl dormancy of cultured herbaceous peony embryos. Plant Cell Tissue Organ Cult..

[26] Yang H., Pei D. (2006). Study on embryo culture of peony (*Paeonia* L.) seed. Guangxi Agric. Sci..

[27] Wang H., van Staden J. (2001). Establishment of in vitro cultures of tree peonies. S. Afr. J. Bot..

[28] Wang Y., He G.M., Han L.X. (2012). Study on embryo-culture and seedling growth for *Paeonia rockii*. Hunan Agric. Sci..

[29] Villiers T.A., Wareing P.F. (1965). The possible role of low temperature in breaking the dormancy of seeds of *Fraxinus excelsior* L.. J. Exp. Bot..

[30] Nyachiro J.M., Clarke F.R., DePauw R.M., Knox R.E., Armstrong K.C. (2002). Temperature effects on seed germination and expression of seed dormancy in wheat. Euphytica.

[31] Penfield S., Josse E.-M., Kannangara R., Gilday A.D., Halliday K.J., Graham I.A. (2005). Cold and light control seed germination through the bHLH transcription factor SPATULA. Curr. Biol..

[32] Derkx M.P.M., Karssen C.M. (1993). Effects of light and temperature on seed dormancy and gibberellin-stimulated germination in *Arabidopsis thaliana*: Studies with gibberellin-deficient and -insensitive mutants. Physiol. Plant.

[33] Taylorson R.B. (2017). Phytochrome controlled changes in dormancy and germination of buried weed seeds. Weed Sci..

[34] Baskin J.M., Baskin C.C. (1986). Seed germination ecophysiology of the woodland herb *Asarum canadense*. Am. Midl. Nat..

[35] Baskin C.C. (2003). Breaking physical dormancy in seeds–focussing on the lens. New Phytol..

[36] Hao H.P., He Z., Li H., Shi L., Tang Y.D. (2013). Effect of root length on epicotyl dormancy release in seeds of *Paeonia ludlowii*, Tibetan peony. Ann. Bot..

[37] Riefler M., Novak O., Strnad M., Schmülling T. (2006). *Arabidopsis* cytokinin receptor mutants reveal functions in shoot growth, leaf senescence, seed size, germination, root development, and cytokinin metabolism. Plant Cell.

[38] Chory J., Reinecke D., Sim S., Washburn T., Brenner M. (1994). A role for cytokinins in de-etiolation in *Arabidopsis* (det mutants have an altered response to cytokinins). Plant Physiol..

[39] Müller K., Tintelnot S., Leubner-Metzger G. (2006). Endosperm-limited Brassicaceae seed germination: Abscisic acid inhibits embryo-induced endosperm weakening of *Lepidium sativum* (cress) and endosperm rupture of cress and *Arabidopsis thaliana*. Plant Cell Physiol..

[40] Ogawa M., Hanada A., Yamauchi Y., Kuwahara A., Kamiya Y., Yamaguchi S. (2003). Gibberellin biosynthesis and response during *Arabidopsis* seed germination. Plant Cell.

[41] Richards D.E., King K.E., Ait-Ali T., Harberd N.P. (2001). How gibberellin regulates plant growth and development: A molecular genetic analysis of gibberellin signaling. Annu. Rev. Plant Biol..

[42] Groot S.P.C., Karssen C.M. (1987). Gibberellins regulate seed germination in tomato by endosperm weakening: A study with gibberellin-deficient mutants. Planta.

[43] Leubner-Metzger G. (2001). Brassinosteroids and gibberellins promote tobacco seed germination by distinct pathways. Planta.

[44] Yamaguchi S., Kamiya Y. (2001). Gibberellins and light-stimulated seed germination. J. Plant Growth Regul..

[45] Amen R.D. (1968). A model of seed dormancy. Bot. Rev..

[46] Yamauchi Y., Ogawa M., Kuwahara A., Hanada A., Kamiya Y., Yamaguchi S. (2004). Activation of gibberellin biosynthesis and response pathways by low temperature during imbibition of *Arabidopsis thaliana* seeds. Plant Cell.

[47] Hennig L., Stoddart W.M., Dieterle M., Whitelam G.C., Schäfer E. (2002). Phytochrome E controls light-induced germination of *Arabidopsis*. Plant Physiol..

[48] Barrero J.M., Jacobsen J.V., Talbot M.J., White R.G., Swain S.M., Garvin D.F., Gubler F. (2012). Grain dormancy and light quality effects on germination in the model grass *Brachypodium distachyon*. New Phytol..

[49] Goggin D.E., Powles S.B., Toorop P.E., Steadman K.J. (2011). Dark-mediated dormancy release in stratified *Lolium rigidum* seeds is associated with higher activities of cell wall-modifying enzymes and an apparent increase in gibberellin sensitivity. J. Plant Physiol..

[50] Long R.L., Stevens J.C., Griffiths E.M., Adamek M., Powles S.B., Merritt D.J. (2011). Detecting karrikinolide responses in seeds of the Poaceae. Aust. J. Bot..

[51] Zhao X., Yu X., Foo E., Symons G.M., Lopez J., Bendehakkalu K.T., Xiang J., Weller J.L., Liu X., Reid J.B. (2007). A study of gibberellin homeostasis and cryptochrome-mediated blue light inhibition of hypocotyl elongation. Plant Physiol..

[52] Jacobsen J.V., Barrero J.M., Hughes T., Julkowska M., Taylor J.M., Xu Q., Gubler F. (2013). Roles for blue light, jasmonate and nitric oxide in the regulation of dormancy and germination in wheat grain (*Triticum aestivum* L.). Planta.

[53] Gubler F., Hughes T., Waterhouse P., Jacobsen J. (2008). Regulation of dormancy in barley by blue light and after-ripening: Effects on abscisic acid and gibberellin metabolism. Plant Physiol..

[54] Goggin D.E., Steadman K.J., Powles S.B. (2008). Green and blue light photoreceptors are involved in maintenance of dormancy in imbibed annual ryegrass (*Lolium rigidum*) seeds. New Phytol..

